# Antibiotic Toxicity Isolated and as Binary Mixture to Freshwater Algae *Raphidocelis subcapitata*: Growth Inhibition, Prediction Model, and Environmental Risk Assessment

**DOI:** 10.3390/toxics10120739

**Published:** 2022-11-29

**Authors:** Fang Chang, Malan Yi, Huiting Li, Jiangnan Wang, Xuefeng Zhao, Xiaoyue Hu, Qianju Qi

**Affiliations:** 1 Marine Resources Research Centre, Tianjin Research Institute for Water Transport Engineering, M.O.T., Tianjin 300456, China; 2 Hanjiang Bureau of Hydrology and Water Resources, Bureau of Hydrology, Changjiang Water Resources Commission, Xiangyang 441000, China; 3 School of Environmental Science and Engineering, Huazhong University of Science and Technology, Wuhan 430074, China

**Keywords:** mixture toxicity, CA and IA models, environmental risk assessment, algae, antibiotics

## Abstract

Antibiotics in aqueous environments can have extremely adverse effects on non-targeted organisms. However, many research projects have only focused on the toxicological evaluation of individual antibiotics in various environments. In the present work, individual and binary mixture toxicity experiments have been conducted with the model organism *Raphidocelis subcapitata* (*R. subcapitata*), and a mixture concentration-response curve was established and contrasted with the estimated effects on the basis of both the concentration addition (CA) and the independent action (IA) models. In addition, different risk assessment methods were used and compared to evaluate the environmental risk of binary mixtures. The toxic ranking of the selected antibiotics to *R. subcapitata* was erythromycin (ERY) > sulfamethoxazole (SMX) > sulfamethazine (SMZ). In general, the conclusion of this study is that the adverse effects of binary mixtures are higher than the individual antibiotics. The CA model and RQ_STU_ are more suitable for toxicity prediction and risk assessment of binary mixtures. This study reveals the potential ecological risks that antibiotics and their mixtures may pose to water ecosystems, thus providing scientific information for environmental quality regulation.

## 1. Introduction

Antibiotics are extremely used for the treatment and prevention of diseases, and have contributed significantly to the improvement of human and animal health [1,2]. In addition, antibiotics have been extensively applied as growth promoters, significantly reducing production costs and improving animal performance and thereby greatly improving the economic benefits of animal husbandry [3]. However, the overuse of antibiotics and their incomplete metabolism in an organism can lead to excretion from the body in their original form [4]. By way of illustration, about 13% of carbamazepine, the most widely used drug in the treatment of epilepsy, is excreted directly into the body in its original form [5]. Traditional wastewater treatment processes are inefficient with respect to removing antibiotics, resulting in large amounts of antibiotics being discharged into the aquatic environment with the concentrations range from ng/L to μg/L [6,7]. The occurrence and pseudo-persistence of antibiotics in water is currently of wide concern due to their high detection rates and concentrations in the environment [8,9].

Algae, as critical primary producers, are found in a wide variety of water bodies in nature, including rivers, lakes and oceans, providing a food source for higher plants and also producing about 50% of the world’s oxygen [8,10]. All of the factors that adversely affect algal growth may cause changes in ecosystem structure [11]. Therefore, algae are recommended as model organisms for toxicity evaluation of pollutants due to the high growth rate of algae and their rapid response to pollutants [12,13]. All chemicals must utilize a regulated toxicological testing procedure to determine potential hazards to the environment prior to obtaining marketing authorization, which is based on the toxicological data obtained when individual chemicals were tested using algae [4]. However, in natural aquatic environments, algae may be exposed to mixtures of antibiotics, and different types of antibiotics may cause discrepant toxicity through synergistic or antagonistic interactions [14].

At present, the toxicity evaluation of compounds relies mainly on the half maximal effective concentration (EC_50_) of the contaminants on model organisms [15]. In general, the evaluation of the effects of individual pollutants on the environment largely depends on the risk quotients (RQs) approach [16,17]. In fact, multiple pollutants in the environment may interact simultaneously with organisms. Hence the toxic effects of compounds are not caused by a single substance in the pollutant, but may be the result of multiple chemicals acting together [18,19], whereas the workload to determine the mixed toxicity of all compounds using experimental methods is huge and unrealistic. Therefore, concentration addition (CA) and independent action (IA) models have been proposed to predict the mixed toxicity of compounds [20,21]. The application of the concept of mixture risk toxicity decreases deviations in the evaluation of mixture toxicity and may predict more accurately the potential effects of antibiotics on aquatic organisms [22].

The green algae *Raphidocelis subcapitata* (*R. subcapitata*) used in this study is widely used worldwide for bioassays in toxicological risk assessments [12]. Sulfamethoxazole (SMX), sulfamethazine (SMZ) and erythromycin (ERY) were used in this study, owing to the widespread utilization and elevated detection rate. The objectives of this study were to: (1) investigate the individual and combined toxicity of three antibiotics on *R. subcapitata* based on standard growth inhibition experiments; (2) utilize CA and IA models to predict toxic effects of binary mixtures, and compare with experimental data; (3) compare the differences in different risk assessment methods; and (4) verify the feasibility of structure-activity relationship (QSAR) in toxicity evaluation and risk assessment.

## 2. Materials and Methods

### 2.1. Algae Culture Procedures

Green algae *R. subcapitata* (FACHB-271) used in this experiment was purchased from the Institute of Hydrobiology, Chinese Academy of Sciences (Wuhan, China), which was cultured under the following conditions: BG11 medium, pH = 7.1 ± 0.1, light intensity of 3000 lux, temperature of 25 ± 2 °C, a light to shade duration ratio of 16:8, and the initial density was 1 × 10^4^ cells/mL. To avoid contamination of the algae species, all inoculation operations were carried out in a vertical flow aseptic station. The Erlenmeyer flasks used for culture were autoclaved at 121 °C. In addition, the algae were constantly shaken in the shaker light incubator during the incubation process to prevent the algae from growing against the walls. To reduce potential experimental errors due to light intensity, Erlenmeyer flasks need to be placed randomly. The Erlenmeyer flaks were sealed with a sterile and breathable membrane during the entire incubation process. For the stability of the experimental results, the algae need to be stabilized and cultured for 30 days before conducting formal experiments to facilitate adaptation to laboratory conditions. The pre-experimental culture conditions were exactly the same as the formal experimental conditions.

### 2.2. Chemicals

SMX (HPLC grade ≥98%) was purchased from Shanghai Yuanye Bio-Technology Co., Ltd. SMZ (HPLC grade ≥ 98%) and ERY (HPLC grade ≥ 98%) were obtained from TCI Europe N.V. (Kawaguchi, Japan). Sulfamethoxazole-^13^C6 (HPLC grade ≥ 99.4%) was obtained from Sigma-Aldrich (St. Louis, MO, USA). Sulfamethazine-d4 (HPLC grade ≥ 99%) and Erythromycin-^13^C, d3 (HPLC grade ≥ 99%) were purchased from J&K Scientific (Beijing, China). Atrazine D5 (HPLC grade ≥ 99%) was commercially purchased from Macklin Biochemical Co., Ltd. Methanol (HPLC grade ≥ 99.9%) and acetonitrile (HPLC grade ≥99.9%) were obtained from Adamas-beta Inc. Ultrapure water was prepared by Ulupure purification system.

### 2.3. Algal Growth Inhibition Test

Range finding assays were conducted prior to the formal experiments to confirm the concentration range in which SMX, SMZ, ERY, and their mixtures (SMX + SMZ, SMX + ERY) might affect *R. subcapitata*. The added antibiotics needed to be prepared as a 10 mg/L stock solution and then gradient diluted to prepare the experimental solution. In the preparation of the stock solution, methanol was used as the co-solvent for antibiotics with low solubility, and the addition of methanol at less than 0.1% (*v*/*v*) to confirm it doesn’t affect algae growth. The stock solution was filtered through a sterile 0.22 μm syringe filter to ensure the sterility of the experimental system. The experimental conditions for conducting algal growth inhibition tests were the same as in Section 2.1, and the duration of the test was based on the OECD 201 guideline [23], with 96 hours as the most commonly used node in toxicology. Based on pre-experiment, the following concentration ranges were established: SMX (0, 0.1, 0.3, 0.5, 0.7, 0.9 and 1.2 mg/L), SMZ (0, 0.5, 1, 2, 4, 6 and 8 mg/L) and ERY (0, 0.01, 0.03, 0.05, 0.07, 0.09 and 0.11 mg/L). The toxicity experiments for binary mixtures (SMX + SMZ, SMX + ERY) were designed according to the classification and mode of action of compounds. The mixed concentrations of binary mixtures were confirmed on the basis of the EC_50_ of individual.

Cell counts were calculated with reference to previous studies with the aid of absorbance at 680 nm [8,24]. In other words, the standard curve between the number of algal cells and absorbance was plotted using a hemocytometer plate and microscope for counting and spectrophotometric measurement of the absorbance of different numbers of algal cell cultures, as the equation below shown:

Cell number (/mL) = 2614.2 OD_680_ − 87.75 (R^2^ = 0.992) (1)

The EC_50_ values of *R. subcapitata* on SMX, SMZ and ERY were calculated by GraphPad Prism 9.4.0. EC_50_ values can be calculated using the log (inhibitor) vs. response-variable slope (four parameters) model based on the relationship between inhibition rate and concentration, as estimated by the following equation [25]:

(2) $$\mathrm{Log}(\mathrm{SMX}\;\mathrm{concentration})={\mathrm{LogEC}}_{50}+(\frac{1}{\mathrm{Hillslope}})\;\times \;\mathrm{Log}\;(\frac{F\%}{100\;-\;F\%})$$

(3) $$F\%=\frac{Y\;-\;\mathrm{Bottom}}{\mathrm{Top}\;-\;\mathrm{Bottom}}\;\times \;100$$

(4) $$Y=\frac{\mathrm{Bottom}+\left(\mathrm{Top}\;-\;\mathrm{Bottom}\right)}{1+10^{\left(\mathrm{LogEC}50\text{-}X\right)}\;\times \;\mathrm{Slope}}$$

where Hillslope means the steepness of curves. Bottom and Top represent the maximal response and basal response, respectively. The Bottom is restricted to 0 according to common sense. F% represents a response between Bottom and Top.

### 2.4. Mixture Toxicity Predictions

CA and IA models are the two most commonly used models when predicting the toxicity of binary mixtures. Among them, the CA model is based on the same mechanism or mode of action between the two compounds, and the equation is shown below [21,26]:

(5) $${\mathrm{ECx}}_{\mathrm{mix}}={\left(\sum_{i=1}^{n}\frac{P_{i}}{{\mathrm{ECx}}_{i}}\right)}^{-1}$$

where ECx_mix_ is the effective concentration at which the mixture results in x% inhibition. P_i_ is the proportion of the mixture when the i-th compound causes x% inhibition. ECx_i_ is the concentration at which compound i, when used alone, leads to x% inhibition.

The IA model is based on the premise that compounds are independent of each other and do not interfere with each other. The Equation for this model is as follows:

(6) $${\mathrm{ECx}}_{\mathrm{mix}}=1-\prod_{i=1}^{n}\left(1-{\mathrm{ECx}}_{i}\right)$$

where ECx_mix_ is the effect caused under the total concentration of the mixture. ECx_i_ is the inhibition effect caused by the corresponding individual pollutant concentration under the total concentration.

### 2.5. Ecotoxicological Risk Assessment

RQs allow predicting the potential ecological impact of contaminants at environmentally relevant concentrations. In the present study, we used two different methods for risk assessment of three antibiotics and their mixtures. The first method, which is currently widely used in risk evaluation, is based on measured environmental concentration (MEC) and predicted no-effect concentration calculations (PNEC). In this case, the MEC uses the highest value that has been detected in the environment. The PNEC value is obtained using the EC_50_ divided by the assessment factor (AF) which is considered as a value of 1000. MEC values are derived from toxicological data in the published literature. The RQs of the mixture (RQ_MEC/PNEC_) are the sum of the RQs of the individual pollutants [5,27]. The calculation is shown in Equations (7) and (8):

(7) $${\mathrm{RQ}}_{\mathrm{MEC}/\mathrm{PNEC}}=\sum_{i=1}^{n}\frac{{\mathrm{MEC}}_{i}}{{\mathrm{PNEC}}_{i}}$$

(8) $$\mathrm{PNEC}={\mathrm{EC}}_{50}/\mathrm{AF}$$

The second method is a computational evaluation based on the toxicity of mixture and is known as RQ_STU_ (STU, the sum of toxic units). As shown in Equation (9), the RQ_STU_ value is usually calculated using three trophic levels (algae, *Daphnia* and fish). In brief, the highest MEC and EC_50_ were used to calculate the STU for each trophic level, after which the maximum STU value was selected and multiplied by AF (1000):

(9) $${\mathrm{RQ}}_{\mathrm{STU}}=\max\left(\sum_{i=1}^{n}\frac{{\mathrm{MEC}}_{i}}{\mathrm{EC}50_{i,\;\mathrm{algae}}},\;\sum_{i=1}^{n}\frac{{\mathrm{MEC}}_{i}}{\mathrm{EC}50_{i,\;\mathrm{Daphnia}}},\sum_{i=1}^{n}\frac{{\mathrm{MEC}}_{i}}{\mathrm{EC}50_{i,\;\mathrm{fish}}}\right)\times \mathrm{AF}$$

### 2.6. Antibiotics Analysis Procedures

Antibiotics concentrations were quantified by liquid chromatography tandem mass spectrometry (LC-MS/MS, Agilent 1290/6460, Santa Clara, CA, America). The samples were pretreated by solid phase extraction prior to LC/MS/MS analysis. During this treatment, internal standards and surrogates need to be added to each sample. Details of the processing procedures and instrumental analyses were provided in Supplementary Information Text S1 and Tables S1 and S2. According to the OECD 2011 guideline [23], initial and final concentrations of antibiotics need to be measured during the 96-hour toxicology test in order to assess the stability of the antibiotics in the test system. Therefore, we set up a non-biological control group (Add antibiotics to BG11 medium without algae) to evaluate the stability of the three antibiotics. Since the light wavelength of our incubator was above 400 nm, the photodegradation was basically negligible. The test data indicated that there was basically no difference between the initial and final concentrations of the three antibiotics. In addition, the difference between the actual and nominal concentrations of the final concentrations was less than 20%. Hence, the nominal concentrations were used for data analysis in this experiment.

### 2.7. Statistical Analysis

In this experiment, three parallel trials were set up for all experimental groups. The one-way analysis of variance and sigmoidal concentration-response curve are implemented using GraphPad Prism 9 (San Diego, CA, USA).

## 3. Results

### 3.1. Single Toxicity Assessments

The fresh algae *R. subcapitata* used in this study was considered as reliable, owing to the validation and acceptability criteria [23]. The 96-h EC_50_ of compounds, which relies on dosage effects, is commonly used for chemical risk evaluation. The relationships between biomass and concentration for SMX, SMZ, and ERY were provided in Figure S1. SMX, SMZ and ERY EC_50_ values at 96 h were calculated by log (inhibitor) vs. response-variable slope (four parameters) model, and R^2^ ≥ 0.9856, 0.9992 and 0.9757 for *R. subcapitata*, respectively, which indicates significant applicability of the model in estimating EC_50_. The EC_50_ values of SMX, SMZ and ERY in this study were 0.6120 mg/L, 3.235 mg/L and 0.056 mg/L, respectively (Table S3). The results indicated that ERY was significantly more toxic to *R. subcapitata* than SMX and SMZ. Similarly, Table 1 showed the published EC_50_ values of SMX, SMZ, and ERY in different green algal species and significant variability was found among the antibiotics. The data collected from the literature revealed that ERY was the most toxic to the algae and SMZ the least toxic, which provides the necessary evidence for this study.

### 3.2. Toxicity Assessments of Binary Mixtures

At present, the ratio of the binary mixture can be selected in two ways: a fixed constant ratio (1:1) or equal ratios of their individual EC_50_ [6,14,25,36]. However, the ratios of disparate antibiotics present considerable difference in a variety of environments. Importantly, the suitability of the model is determined based on the type of mixture, whereas the ratio of the mixture is selected on account of the value of EC_50_. In the present work, we are focusing on the applicability of CA and IA models in predicting the joint effect of binary mixture. The CA and IA models are based on the premise that the relationship of interactions. Therefore, we selected SMX + SMZ and SMX + ERY as the representative of same and independent mechanism, respectively, to verify the reliability of CA and IA models in toxicity prediction and risk assessment. The growth inhibition of *R. subcapitata* under SMX + SMZ and SMX + ERY mixtures stress was presented in Table 2.

**Figure 1:**
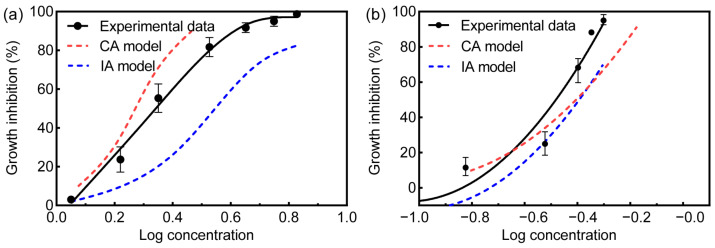
The comparison of experimental data ((**a**) SMX + SMZ and (**b**) SMX + ERY) and the predicted dose-response curves. Error bars represent SD ± mean.

Considering the EC_50_ values, the mixture of SMX + SMZ and SMX + ERY presented significant toxic effects than the sum of the EC_50_ values of the individual substances (Table S3 and Table 2). The prediction curves were established on the basis of IA and CA models for assessing the toxicity of binary mixtures (Figure 1). The simulative model deviation ratio (MDR), a ratio of experimental data to model data, was used to evaluate deviation throughout the dataset, which was defined as perfect fit (MDR = 1), underestimation (MDR < 1) and overestimation (MDR > 1). The model was considered reliable whilst the interval of MDR values was between 0.5 and 2. According to the experimental data, the EC_50_ value of the mixture of SMX + SMZ towards *R. subcapitata* was determined to be 2.146 mg/L. By contrast, the predicted EC_50_ values of SMX + SMZ by the CA and IA models were 1.864 mg/L and 3.253 mg/L, respectively (Table 2). The MDR values of CA and IA models were 1.151 and 0.6597, respectively. Similarly, the EC_50_ values of SMX + ERY was 0.0.3716 mg/L, whereas the predicted EC_50_ values of CA and IA models were 0.4077 mg/L and 0.4267 mg/L, and the MDR values of CA and IA models were 0.9115 and 0.8709, respectively. In the present work, the MDR values range from 0.58 to 1.623, which indicates that the CA and IA models are suitable for predicting the toxicity of binary mixtures.

### 3.3. Environmental Risk Assessment

**Figure 2:**
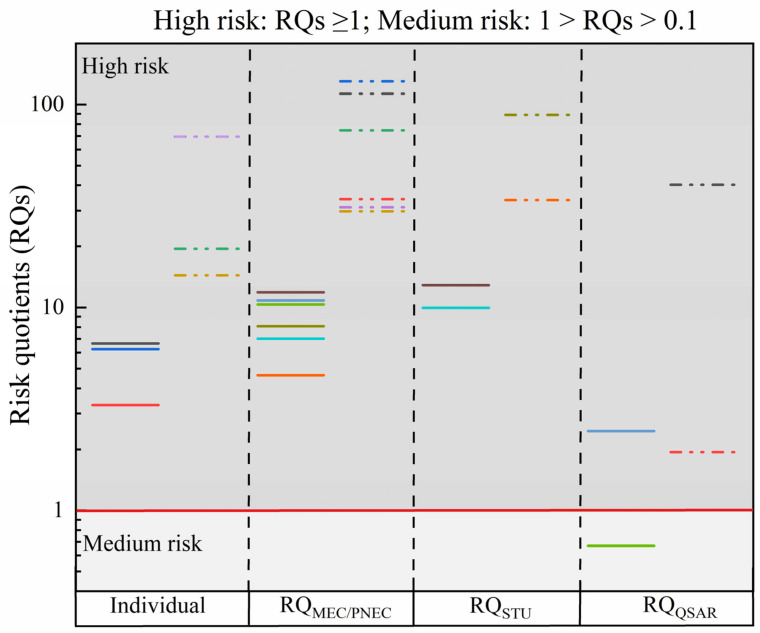
RQs for individual and binary mixtures obtained based on different environmental risk assessment methods. The solid line and dotted line represent the RQs in surface water and wastewater, respectively.

The risk quotient approach is the method that the Environmental Fate and Effects Division (EFED) applied to integrate exposure results and ecotoxicity data [37], which is able to effectively combine laboratory toxicological data and environmental risks to comprehensively evaluate the risk level of contaminants in the natural environment. According to RQs value, the ecological risk ranking criterion of antibiotics was defined as four grades: RQs < 0.01, insignificant risk; 0.01 ≤ RQs < 0.1, low risk; 0.1 ≤ RQs < 1, medium risk; and RQs ≥ 1, high risk [38]. In the present work, the MEC values for SMX, SMZ, and ERY in surface water and wastewater were used to evaluate the negative effects of SMX, SMZ, ERY and binary mixtures for the aquatic environment, and the RQs values were presented in Figure 2 and Table S4. The environmental risks of SMX, SMZ, ERY, and binary mixtures were defined as high risk on account of the RQs values greater than 1, indicating a greater ecological risk for the three tested antibiotics at relatively high environmental concentrations. In addition, the RQs values of antibiotics in wastewater presented much higher than that in surface water. Similarly, RQ_STU_ was applied to evaluate risks, leading to similar conclusions. The RQ values of mixtures assessed by both methods were higher than those of individual compounds, both in surface water and wastewater, confirming that these antibiotic mixtures pose a more serious ecotoxic risk and widespread contamination to the aquatic environment and are of concern. QSAR is appropriate to predict the toxicity of pollutants on trophic level specific (e.g., algae, *Daphnia*, and fish) [39,40], which coincides with the calculation process of RQ_STU_. In this study, the EC_50_ for different trophic levels predicted using the model is shown in Table S4 and Figure 2. The RQ_QSAR_ values of SMX + SMZ (based on QSAR and STU) calculated based on algae, *Daphnia*, fish are 0.751, 2.46 and 0.072, respectively. Similarly, the RQ_QSAR_ values of SMX + ERY are 0.242, 0.674, and 0.0664, respectively. QSAR was used for environmental risk assessment to obtain a lower risk level than RQ_STU_ and RQ_MEC/PNEC_.

## 4. Discussion

The green algae *R. subcapitata* is continually used in environmental regulations and toxicity assessment of chemicals, which need vast amounts of data to satisfy the requirements [41,42]. However, due to the wide variety of chemicals, the task of testing all of them for toxicity is almost impossible to accomplish [43]. At present, traditional toxicology experiments tend to use individual antibiotics to evaluate the toxicity and hazards of antibiotics. In previous studies, SMX interferes with chlorophyll synthesis and photorespiration capacity, affecting DNA replication and repair, which in turn affects the growth of *R. subcapitata* [44]. SMX and SMZ induced oxidative stress in algal cells and interferes with cell growth [8,33]. ERY inhibited the protein synthesis in chloroplasts and mitochondria, which results in a disturbance of photosynthetic and mitochondrial activity [45]. In addition, it was accompanied by a physiological phenomenon of increased autofluorescence and reduction of chlorophyll a content whilst algae exposed to ERY [45]. Similarly, the redox homeostasis and antioxidant system of *Pseudokirchneriella subcapitata* (*Raphidocelis subcapitata*) was affected by toxicants, which leads to lipid peroxidation and algal cell membrane damage [46,47]. On the other hand, the antibiotics have been listed in priority substances and the Directive 2013/39/EU [48] to stress the need for a strategic approach to antibiotic contamination. As a consequence, the toxicity and environmental risk of mixed antibiotics, especially binary mixtures to aquatic organisms, represents a constant concern [36,49,50]. According to EU Directive 93/67/EEC [51], the chemical substances were defined as different categories on account of EC_50_ values: 10–100 mg/L, harmful; 1–10 mg/L, toxic; <1 mg/L, very toxicity. Therefore, SMX and ERY could be classified as very toxic substances and SMZ as toxic substances. However, the environmental risk of SMZ in wastewater presented considerably higher than SMX and ERY owing to elevated residual concentrations.

Aquatic organisms in realistic environments may be exposed to mixtures of different chemicals [52,53], hence binary mixtures were used to obtain more veritable information about the possible effects of antibiotic mixtures on *R. subcapitata* [8]. Generally, the toxicity resulting from the coexistence of two antibiotics in the natural environment is greater than that of individual chemicals, causing more severe adverse biological effects [54,55]. The predictive performance of CA and IA models in binary mixtures is adequately presented owing to the MDR values. In this study, the experimental results demonstrated that the CA model overestimated the toxicity of SMX + SMZ and SMX + ERY mixtures, while the IA model underestimated toxicity levels. A previous study has reported that CA models are more suitable for estimating the combined toxicity of mixtures of pharmaceuticals (Ibuprofen, Ciprofloxacin and Chlorophenols) to *C. vulgaris* [25,36]. Likewise, in the case of SMX + SMZ, the difference in values between the CA model and experimental data presented less than the difference between the IA model and experimental data, which denotes that CA model shows superior performance in terms of toxicity prediction. However, in term of SMX + ERY, the CA model is suitable for toxicity prediction of mixing at low concentrations while the IA model shows excellent applicability for toxicity prediction of mixing at high concentrations. The experimental results might reveal that the change is related to a smaller number of pathways or targets activated, which could be attributed to one of the compounds playing a leading role in this change. On the contrary, the IA model is suitable for low concentration mixtures when 2,4-dichlorophenol and ibuprofen were mixed, while the CA model is appropriately applied to high concentration mixtures [25]. Therefore, based on the experimental results and previous studies, we inferred that the selection of prediction model depends upon the biochemical mode of action (MOA) of chemicals. However, the MOA information is scarce and insufficient for supporting error-free model selection. In the vast majority of cases, the toxicity predicted using CA models is usually higher than IA. The model predicts the worst-case scenarios for risk assessment, and represent a more widely used method in toxicity evaluation [56,57]. Additionally, the combined effects of chemicals which possess different MOA can also be predicted by CA [58].

The ecological risk of a contaminant is related to its inherent toxicity and is also influenced by the residual concentration in the environment [59]. Therefore, the ecotoxicity assessment of mixtures using different evaluation methods in combination with the actual concentration of antibiotics in the environment is beneficial to comprehensively assess the environmental effects of contaminants [60]. Previous studies have focused on RQs calculation was simplified by using individual chemicals and/or the sum of individual RQs [8,25,61]. In this study, the RQ values for individual antibiotics and binary mixtures revealed that SMX, SMZ, ERY, SMX + SMZ, and SMX + ERY were higher than the threshold value, indicating a potentially high ecological risk to the aquatic environment. In particular, the risk levels of mixed antibiotics in wastewater were much higher than the critical threshold. Although for wastewater containing pollutants the final environmental risk is related to the degree of dilution in surface water [62], the adverse environmental effects caused by their frequent discharges into the environment also need to be considered [63]. The RQ caused by 3847 ng/L of ERY was reported to be as high as 18.77 in the stowed water of a Spanish wastewater treatment plant [64]. Incomplete removal of SMX, trimethoprim, azithromycin, and clarithromycin from wastewater poses a high risk to aquatic organisms in the receiving environment as the RQ values all exceed 10 [65,66]. Similarly, the RQ_STU_ values of binary mixtures exceeds the threshold of high risk, the difference between RQ_STU_ and RQ_MEC/PNEC_ is insignificant, and the RQ_STU_ of wastewater is higher than that of surface, which can be attributed to the high concentration of SMZ in wastewater. In addition, The QSAR for environmental risk assessment underestimates the adverse environmental effects of mixtures. Moreover, the toxicological data obtained from the QSAR model and our experimentally obtained data have some deviations, which are not absolutely convincing.

This work revealed the toxicity of individual and binary mixtures by CA, IA models, and environmental risk by RQ_STU_, RQ_MEC/PNEC,_ and RQ_QSAR_. However, the method of risk calculation using traditional endpoints and standard species has certain limitations. In the future, the comprehensive ecological risk evaluation model needs to be refined so that the environmental risks of mixtures can be productively evaluated. Additionally, there is an urgent need to supplement long-term toxicity data to facilitate chronic studies over the biological life cycle, providing a basis for chronic and mixed toxicity of compounds at low concentrations.

## 5. Conclusions

In the present work, the toxicity of binary mixtures was precited using CA and IA models, and the environmental risk assessment was evaluated by RQ_STU_ and RQ_MEC/PNEC_. Compared with the IA model, the CA model presented eminent feasibility, owing to the CA model possessing a smaller gap with the experimental data upon most occasions. In addition, the CA model provided higher toxicity data, which is conducive to providing higher standard supporting data for environmental protection. Based on the residual concentration in the environment, the environmental risk assessment of binary mixtures possesses insignificant difference. However, the RQ_STU_ data obtained based on trophic level can more veritably reflect the adverse effects of binary mixtures on the environment.

## Figures and Tables

**Table 1:** The ecotoxicological effects of SMX, SMZ, and ERY on the growth inhibition of microalgae were summarized and contrasted with previously reported data.

Antibiotics	Algal Species	EC_50_ (mg/L)	Endpoint and Duration of Test	References
SMX	*R. subcapitata*	4.74	96 h	[28]
SMX	*R. subcapitata*	0.52	72 h	[29]
SMX	*C. vulgaris*	1.51	72 h	[30]
SMX	*R. subcapitata*	0.612	96 h	This study
SMZ	*R. subcapitata*	7.8	72 h	[31]
SMZ	*R. subcapitata*	8.7	72 h	[32]
SMZ	*C. vulgaris*	31.35	96 h	[33]
SMZ	*R. subcapitata*	3.235	96 h	This study
ERY	*R. subcapitata*	0.0246	96 h	[34]
ERY	*R. subcapitata*	0.038	72 h	[35]
ERY	*R. subcapitata*	0.044	96 h	[8]
ERY	*C. vulgaris*	0.36	96 h	[31]
ERY	*R. subcapitata*	0.056	96 h	This study

**Table 2:** Model deviation ratios (MDRs) values at different growth inhibitory concentrations for binary mixtures for *R. subcapitata*.

Growth Inhibition	SMX + SMZ					SMX + ERY				
(%)	ED ^a^	CA	CA-MDR ^b^	IA	IA-MDR ^c^	ED ^a^	CA	CA-MDR ^b^	IA	IA-MDR ^c^
10	1.283	1.185	1.082	1.711	0.75	0.2534	0.1561	1.623	0.3260	0.7773
30	1.760	1.572	1.120	2.539	0.6932	0.2907	0.2807	1.036	0.3206	0.9067
50	2.146	1.864	1.151	3.253	0.6597	0.3716	0.4077	0.9115	0.4267	0.8709
70	2.617	2.226	1.176	4.168	0.6278	0.4308	0.4921	0.8754	0.4734	0.91
90	3.589	2.932	1.224	6.186	0.58	0.5451	0.669	0.8148	0.5585	0.976

^a^ ED represents experimental data. ^b^ CA-MDR represents a ratio of experimental data to CA model data. ^c^ IA-MDR represents a ratio of experimental data to IA model data.

## Data Availability

The data materials are shown in the main text and can also be acquired upon request from the corresponding author.

## Supplementary Materials

The following supporting information can be downloaded at: https://www.mdpi.com/article/10.3390/toxics10120739/s1, Figure S1: Effect of (a) SMX, (b) SMZ and (c) ERY on the biomass of *R. subcapitata*. * represents a statistically significant difference (*p* < 0.05), Table S1: Instrumental analysis of LC-MS/MS; Table S2: Optimized retention time, ion transitions, ion transitions, collision cell exit potential for MS/MS determination of target antibiotic; Table S3: Parameters of *R. subcapitata* at 96 h were fitted using log (inhibitor) and response-variable slope (four parameters) models; Table S4: Effective concentration (EC_50_) and risk quotients of SMX, SMZ, ERY and their mixture for *R. subcapitata*, Text S1. The method of solid phase extraction and LC-MS/MS.
