# Supplementary material for: Antibiotic Toxicity Isolated and as Binary Mixture to Freshwater Algae Raphidocelis subcapitata: Growth Inhibition, Prediction Model, and Environmental Risk Assessment
Source: Toxics. 2022 Nov 29;10(12):739. doi: 10.3390/toxics10120739 (PMC9785756; doi:10.3390/toxics10120739)
Supplement: Supplementary file 1 [file toxics-10-00739-s001.zip › toxics-2035831-supplementary.pdf]

toxics MDPI

Antibiotic Toxicity isolated and as Binary mixture to Freshwater Algae *Raphidocelis subcapitata*: Growth Inhibition, Prediction Model, and Environmental Risk Assessment

Fang Chang [1], Malan Yi [1], Huiting Li [1], Jiangnan Wang [1], Xuefeng Zhao [2], Xiaoyue Hu [2], Qianju Qi [1,3,*]

Text S1. The method of solid phase extraction and LC-MS/MS

The culture medium was filtered with 0.45 μm micron nylon membrane, and diluted to 50 ml. The 30 ng of internal surrogate standard was added into sample. Then the sample solution was extracted solid phase extraction (SPE), with flow rate of 4–8 ml/min, rinsed the beakers by 10 ml ultra-pure water after samples were all passed through the cartridges, and then evacuated for one hour. The analyte was eluted with acetonitrile and dried in a 37°C water bath under a nitrogen stream. The samples of different concentration were reconstituted with different amounts of methanol. Then added different volumes of Atrazine-d5 internal standards (IS) to these groups, and made the concentration of Atrazine-d5 in each sample is 20 ng/ml. The sample solution was then transferred to an autosampler vial (with insert) for LC/MS/MS analysis.

Supplementary Table S1. Instrumental analysis of LC-MS/MS

**Column**	Agilent ZORBAX Eclipse Plus C18 HPLC column (3 × 100 mm, 1.8 μm)	
**Mobile phase**	A: ultrapure water with 0.1% formic acid (v/v)<br>B: acetonitrile	
Column tem.	24 °C	
Injection volume	10 μL	
Flow rate	0.3 mL/min	
Gradient	A (%)	B (%)
0.00 min	92.5	7.5
1.00 min	92.5	7.5
3.00 min	88.0	12.0
4.50 min	80.0	20.0
6.00 min	40.0	60.0
9.00 min	10.0	90.0
10.00 min	10.0	90.0
11.00 min	92.5	7.5

Supplementary Table S2. Optimized retention time, ion transitions, ion transitions, collision cell exit potential for MS/MS determination of target antibiotic.

Compound	Retention time (min)	Ion transitions (m/z)	Collision energy (eV)	Collision cell exit potential (V)
Sulfamethoxazole	7.06	254 > 155.9	80	15
		254 > 108		25
Sulfamethazine	4.2	275.1 >186.1	120	12
		275.1 >156.1		14
Sulfamethazine-d4	7.01	283.1 > 124.2	124	30
		283.1 > 186.1		20
Atrazine-D5	8.72	221.0 > 101.0	113	30
		221.0 > 137.0		25
		221.0 > 179.0		20
Erythromycin	7.65	734.5 > 158.2	140	30
		734.5 > 576.4		20
Erythromycin-13C-d3	7.31	738.6 > 162.0	146	34
		738.6 > 582.5		19

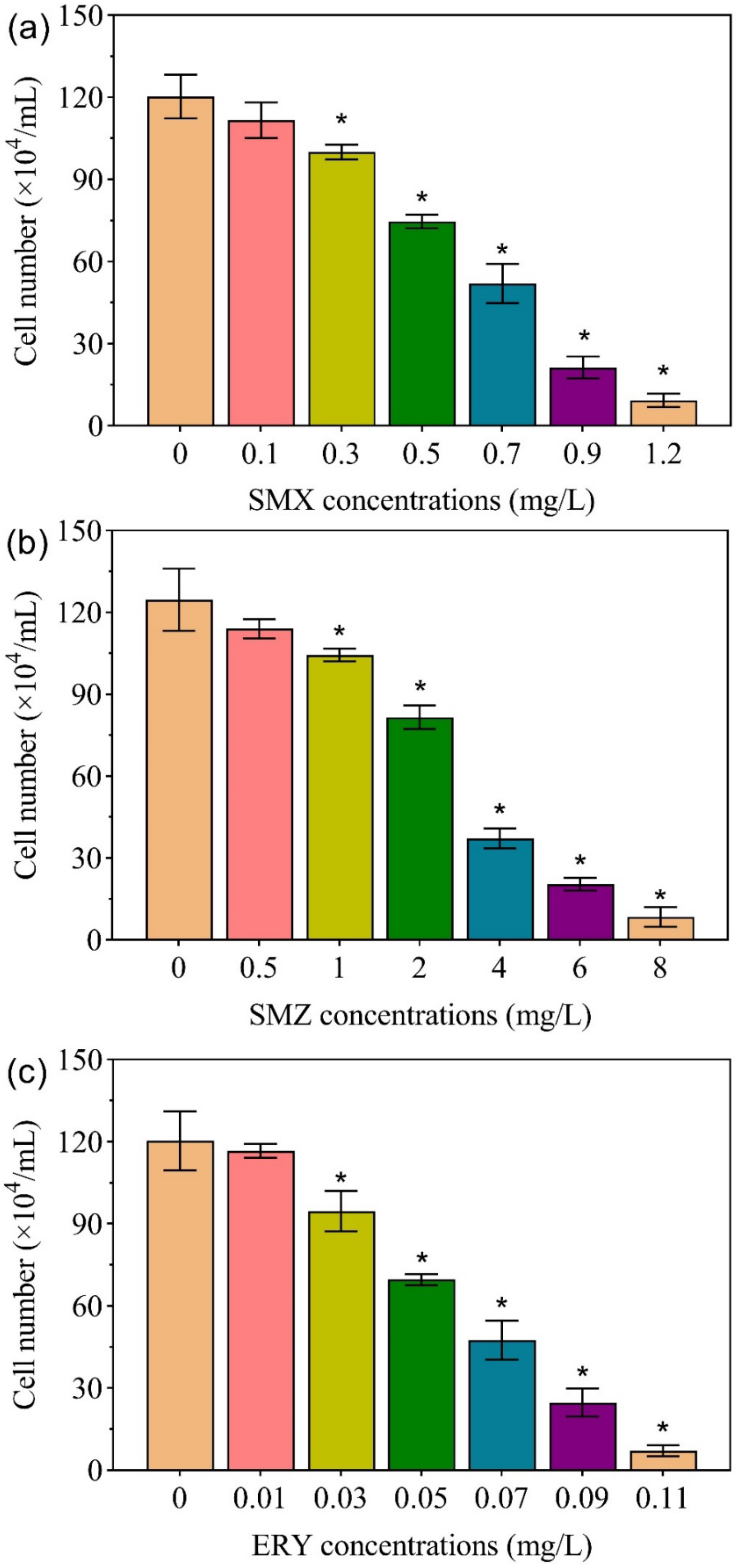

Figure S1. Effect of (a) SMX, (b) SMZ and (c) ERY on the biomass of *R. subcapitata*. * represents a statistically significant difference ($p < 0.05$).

Supplementary Table S3. Parameters of *R. subcapitata* at 96 h were fitted using log (inhibitor) and response-variable slope (four parameters) models.

**Parameters**	**Best-fit values**					**95 % confidence intervals**				
	**SMX**	**SMZ**	**ERY**	**SMX+ERY**	**SMX+SMZ**	**SMX**	**SMZ**	ERY	SMX+SMZ	SMX+ERY
**Top**	114.4 mg/L	96.37 mg/L	117.5 mg/L	114.2	96.74	108.6 to 120.4	87.09 to 114.7	111.3 to 124.0	77.95 to N/A	91.47 to 103.3
**LogEC50**	-0.2132	0.5098	-1.250	-0.4299	0.3316	-2.248 to 2.881	-0.4771 to 0.5560	-1.291 to 1.213	-0.5161 to N/A	0.3025 to 0.3661
**EC50**	0.6120 mg/L	3.235 mg/L	0.056 mg/L	0.3716	2.146	0.5646 to 0.6586	3.000 to 3.597	0.05119 to 0.06126	0.3047 to N/A	2.007 to 2.323
**Span**	114.4	90.62	117.5	114.2	96.74	/	/	/	/	/
**Hill Slope**	-3.320	5.704	-2.785	5.736	4.271	-4.157 to 2.672	-3.683 to 8.509	-3.452 to 2.265	-1.331 to N/A	3.264 to 5.720
**Degrees of Freedom**	18	2	18	3	4	/	/	/	/	/
**R squared**	0.9757	0.9992	0.9756	0.9833	0.9967	/	/	/	/	/
**Sum of Squares**	853.0	5.823	877.9	136.4	28.94	/	/	/	/	/
**Sy.x**	6.884	1.706	6.984	6.743	2.690	/	/	/	/	/

Supplementary Table S4. Effective concentration (EC_{50}) and risk quotients of SMX, SMZ, ERY and their mixture for *R. subcapitata.*

		SMX	SMZ	ERY	SMX+SMZ	SMX+ERY	CA (SMX+SMZ)	IA (SMX+SMZ)	CA (SMX+ERY)	IA (SMX+ERY)
RQ of individual	Surface water	6.650	3.308	6.226	/	/	/	/	/	/
	Wastewater	19.44	69.47	14.41	/	/	/	/	/	/
$RQ_{MEC/PNEC}$ [a]	Surface water	/	/	/	7.022	11.89	8.085	4.633	10.84	10.35
	Wastewater	/	/	/	34.59	1.07	130.32	74.67	31.17	29.79
RQ_{STU} [b]	Surface water	/	/	/	9.959	88.92	/	/	/	/
	Wastewater	/	/	/	12.88	33.85	/	/	/	/
RQ_{QSAR} [c]	Surface water	/	/	/	2.460	40.22	/	/	/	/
	Wastewater	/	/	/	0.6736	1.945	/	/	/	/

[a] The maximum concentration of SMX used to calculate the RQ of surface water and wastewater was 4.07 and 11.9 μg/L, respectively. The maximum concentration of SMZ used to calculate the RQ of surface water and wastewater was 11 and 231 μg/L, respectively. The maximum concentration of ERY used to calculate the RQ of surface water and wastewater in Table 1 was 0.35 and 0.81 μg/L, respectively.

b The EC50 values of SMX, SMZ and ERY to Daphnia were 127.45, 194.8 and 45.1 mg/L, respectively. The EC50 values of SMX, SMZ and ERY to Daphnia were more than 1000 mg/L, which was defined as N/A.

c Based on the QSAR, the EC50 values of SMX, SMZ and ERY to algae were 21.8, 19.5 and 6.37 mg/L, respectively. the EC50 values of SMX, SMZ and ERY to Daphnia were 6.43, 6.02 and 8.62 mg/L, respectively. the EC50 values of SMX, SMZ and ERY to fish were 267, 195 and 6.84 mg/L, respectively.
